# 多发性骨髓瘤患者门诊管理标准流程中国专家共识（2025年版）

**DOI:** 10.3760/cma.j.cn121090-20250123-00044

**Published:** 2025-04

**Authors:** 中华医学会血液学分会浆细胞疾病学组 中国医药教育协会血液学专业委员会

## Abstract

随着新型药物和治疗手段的出现，多发性骨髓瘤（MM）患者的生存期明显延长，MM的治疗已进入慢性病管理阶段。为助力MM的管理，中华医学会血液学分会浆细胞疾病学组和中国医药教育协会血液学专业委员会编写此共识，从建立门诊管理的条件、患者的规范化收治标准及流程、门诊随访管理及患者教育等方面提出建议，旨在建立并优化MM治疗的门诊管理模式，提供更高效的患者管理和随访，从而建立更便捷的诊疗模式。

## Abstract (en)

With the development of new drugs and treatments, the survival of patients with multiple myeloma（MM）is significantly prolonged, and MM treatment has entered the stage of chronic disease management. To assist in MM management, the Plasma Cell Disease Group of the Chinese Society of Hematology and the Hematology Committee of the Chinese Medicine Education Association prepared this expert consensus. It provides recommendations on the conditions of establishing outpatient management, standards and processes of patient treatment, outpatient follow-up management, and patient education. This consensus aims to establish and optimize the outpatient management model of MM treatment, enhance the efficacy of patient management and follow-up, and thereby to establish a more convenient diagnosis and treatment model.

随着新型药物和治疗手段的出现，多发性骨髓瘤（MM）患者的生存期明显延长，MM的治疗已进入慢性病管理阶段。建立并优化MM治疗的门诊管理模式不仅可助力MM的慢性病管理，提供更高效的患者管理和随访，同时还能更合理地分配门诊与住院病房的医疗资源，建立更便捷的诊疗模式。

一、MM患者门诊管理的建议条件

1. 医疗机构的资质：

（1）开展门诊诊治MM患者的医疗机构应具有相应的诊疗能力。

（2）医疗机构门诊应配备抗肿瘤药物的静脉配置场所，并由专人配制药物。

2. 硬件配置：需具备相对独立区域、固定床位或输液椅、护理区域、医护人员办公区。

（1）治疗区位置相对独立，按功能划分为患者出入区域：导医台、等候区、治疗室等；患者禁入区域：药物配置室、消毒室、污洗区等。

（2）按诊疗需求设置床位或输液椅及各类医疗仪器和药品。

（3）设置需要进行急救、抢救（如过敏性休克等）的抢救区域，配置抢救设备及药品。

3. 人员设置和职能建议要求：

（1）人员配备需满足医疗安全要求，医护人员应接受相关知识与专业技能的培训及考核。

（2）可以与相关科室建立多学科团队（Multi-disciplinary team, MDT）诊疗模式，或按照医院规定完成科室间会诊、接诊及转诊。涉及科室包括血液科、肿瘤科、检验科、影像科、疼痛科、肾病科、骨科及医院管理部门等。

（3）可以配备随访团队，包括主诊医师、护士等，明确随访周期、随访事项等。

（4）可以配备患者教育团队，针对疾病知识、药物不良反应、家庭护理及注意事项等定期组织宣讲。

二、门诊管理的规范化收治标准及流程

（一）患者收治标准

制定可以在门诊接受治疗的MM患者标准，如患者存在以下任何1种情况，则应判定为不符合接受门诊治疗，不应于门诊收治：

1. 合并严重并发症和（或）处于感染活动期；

2. 肿瘤负荷高，经主诊医师评估治疗期间可能出现严重并发症；

3. 治疗方案含静脉输注药物且输注时间较长（输注时间>8 h）；

4. 依从性差，沟通能力差，不能充分配合；

5. 生活不能自理且没有具备良好护理能力的陪护人员；

6. 有严重精神疾病或认知障碍，不能配合诊疗；

7. 其他由医师判断不适合门诊治疗的患者。

（二）患者收治流程

1. 诊疗前：

（1）初步评估患者是否符合接受门诊诊疗的条件，并与患者及家属充分沟通病情。

（2）诊前筛查：由主诊医师负责完善必要的相关检查，当日接诊医护人员再次评估患者各项检查结果，避免在存在禁忌证的情况下进行治疗。

2. 诊疗中：

（1）疾病评估相关检查建议：见表1。

**表1:** 不同治疗阶段多发性骨髓瘤患者检查事项建议

检查项目	初诊	诱导治疗期间疗效评估	维持治疗	复发难治阶段
血液检查及频率	首次	每1～2个疗程1次	每3～6个月1次	出现疾病变化时
血常规	推荐	推荐	推荐	推荐
肝肾功能（包括白蛋白、乳酸脱氢酶、尿酸）	推荐	推荐	推荐	推荐
血清蛋白电泳、免疫固定电泳	推荐	推荐	推荐	推荐
血清免疫球蛋白定量（包括轻链）^a^	推荐	推荐	推荐	推荐
β_2_-微球蛋白	推荐	不要求	不要求	推荐
电解质（包括钙离子）	推荐	推荐	推荐	推荐
凝血功能	推荐	不要求	不要求	推荐
心肌酶、肌钙蛋白、NT-proBNP^b^	推荐	建议	建议	推荐
C反应蛋白	推荐	建议（怀疑感染）	建议（怀疑感染）	推荐
尿液检查及频率	首次	每1～2个疗程1次	每3～6个月1次	出现疾病变化时
24 h尿M蛋白定量	推荐	推荐	推荐	推荐
尿免疫固定电泳	推荐	推荐	推荐	推荐
骨髓检查及频率	首次	–	–	出现疾病变化时
骨髓细胞学涂片分类	推荐	推荐^c^	不要求	推荐^d^
骨髓活检+免疫组化	推荐	不要求	不要求	推荐
流式细胞术	推荐	建议	不要求	推荐
细胞遗传学检查	推荐	建议	不要求	推荐
影像学检查及频率	首次	如基线存在浆细胞瘤，2～3个月1次CT检查；如不存在，则不要求	–	出现疾病变化时
局部或全身CT	可选	不要求	出现症状时	可选
全身或局部MRI	可选	不要求	出现症状时	可选
PET-CT	可选	可选^e^	出现症状时	可选
其他检查				
腹壁皮下脂肪、骨髓或受累器官活检+刚果红染色	怀疑淀粉样变性者	不要求	不要求	怀疑淀粉样变性者
超声心动图±心脏MRI	怀疑心功能不全及合并心脏淀粉样变性者	–	–	怀疑心功能不全及合并心脏淀粉样变性者

**注** NT-proBNP：N末端脑钠肽前体；PET-CT：正电子发射计算机断层显像；–：无；^a^轻链型疾病患者必须监测游离轻链；^b^心功能不全及怀疑合并心脏淀粉样变性或轻链沉积病患者，检测肌钙蛋白；^c^完全缓解的患者为推荐，不分泌型建议2～3个月1次；^d^疾病进展患者为推荐；^e^应用PET-CT以确认微小残留病阴性

（2）治疗方案：化疗方案、药物选择等参考最新诊疗指南或高证据级别大型临床研究结果，并结合患者的耐受度和便利性。诱导治疗期间门诊给药优先选择含口服药物（如伊沙佐米、来那度胺等）和（或）皮下注射类药物（如硼替佐米、达雷妥尤单抗等）的方案。有造血干细胞移植计划的患者应考虑治疗方案对造血干细胞采集的影响。维持治疗期间治疗方案应综合患者的疾病特点、长期耐受度、安全性及依从性，优先选择蛋白酶体抑制剂伊沙佐米、来那度胺等口服药物。推荐不伴高危因素的患者至少维持治疗2年，伴高危因素的患者持续维持治疗至疾病进展。

（3）不良事件管理：

①不良事件预防：

• 对于合并基础疾病的患者，应在基础疾病稳定的前提下接受抗肿瘤治疗，同时治疗过程中应增加心、肺、肝、肾等重要脏器功能的监测频率。

• 有血栓高危因素如接受免疫调节剂治疗及深静脉置管的患者，应进行静脉血栓栓塞风险评估，并根据发生血栓栓塞的危险因素予分层预防性抗凝治疗。

• 合并心血管基础疾病者、存在心脏淀粉样变及治疗方案中含心脏毒性药物者均需密切监测生命体征及心力衰竭、容量负荷过重风险，警惕心血管不良事件，及时对症治疗。

• 若使用大剂量地塞米松方案，可考虑预防卡氏肺孢子菌肺炎和真菌感染；使用蛋白酶体抑制剂、达雷妥尤单抗的患者应预防性使用抗病毒药物。

• 对于易发生过敏反应或输注相关反应的药物，尤其是达雷妥尤单抗，可参照药品说明书在治疗前予抗组胺药、退热剂和糖皮质激素等，以降低输注相关反应的发生风险。输注过程中应密切监测，及时予对症治疗。对于接受免疫调节剂（如来那度胺、沙利度胺等）治疗的患者，既往使用时发生严重过敏或4级皮疹的应避免再次使用，如发生2～3级皮疹应考虑暂停或停止用药。接受硼替佐米治疗或初诊即存在外周神经病变患者考虑神经系统专科检查。

• 维持治疗方案含有糖皮质激素者，监测血糖并警惕骨质疏松症。

②常见不良事件的处理原则：

• 抗肿瘤治疗中出现恶心、呕吐、腹泻等消化道不良反应较常见，应积极予止吐、止泻等对症支持治疗，同时向患者及家属进行解释以减轻紧张情绪。

• 定期监测血常规，持续存在症状性贫血的患者可考虑使用促红细胞生成素治疗，酌情补充铁剂、叶酸、维生素B_12_等造血原料，必要时输血治疗。

• 如出现漏服或错服药物，需及时与主诊医师联系，错服药物后出现任何不适应尽快就诊。

• 任何治疗相关不良事件及并发症经积极处理仍不缓解，应经绿色通道尽快转至病房接受治疗。

③急危重症的应急处理：

• 门诊应急转入病房通道的建立：制定急危重症的应急处理预案及不良反应应急处理流程，建立从门诊转至病房的绿色通道。

• 门诊患者如出现药物过敏、输液反应、腹痛、高热等治疗相关不良反应，主诊医师应立即停止治疗并对症处理，如症状缓解，继续观察至症状消失后进行下一步诊疗；如处理后症状持续，则经绿色通道转入普通病房；如症状加重并出现休克、意识改变等，主诊医师立即主持抢救，必要时经绿色通道转至普通病房或ICU进一步治疗。如出现突发心脑血管事件如心肌梗死、心律失常、脑卒中、肺栓塞、气胸、消化道穿孔等危重情况，由主诊医师立即联系急诊ICU，启动紧急救治，联合相关科室会诊，确保患者得到及时有效的救治，必要时转入ICU病房或相关科室继续治疗。

MM患者门诊管理流程见图1。

**图1:**
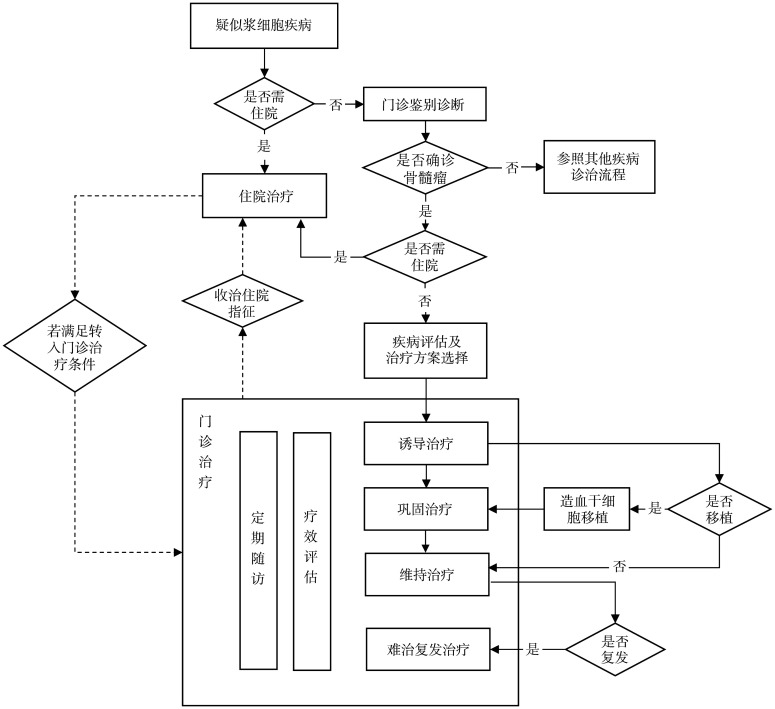
多发性骨髓瘤患者门诊管理流程图

3. 诊疗结束离院前：

（1）嘱咐患者注意观察自身反应，可详细记录并及时向主管医师反馈。

（2）嘱咐患者如果在院外出现严重不良反应，及时到医院就诊以免延误病情。

（三）患者的数据管理

在门诊接受化疗的病历记录应包括初始诊断及相关化验结果、治疗药物及剂量、疗效评估及相关指标、是否出现不良反应等，建议设立独立的数据收集系统如门诊追踪卡/门诊档案/患者卡片等。经治医师及护士应于规定时限内及时完成诊疗内容的记录、知情同意书等医疗文书的存档。

三、MM患者的门诊随访管理

1. 门诊随访由主诊医师作为负责人，由经治医疗小组负责完成，经治小组可包括主诊医师、随访护士或经过培训的协调人员。

2. 随访目的是观察患者病情、处理不良反应，并加强宣教以保证医疗安全。设置服务电话或微信平台或线上门诊以提供咨询服务，保障患者居家安全。

3. 根据疾病阶段及具体情况确定随访内容，如常规复查可至医院普通门诊或便民门诊；如出现治疗相关不良反应，可指导其于附近医院治疗；若症状较重或可能危及生命需入院治疗时，应联系主诊医师安排收治入院。

4. 随访周期：

（1）诱导治疗初期可以每1～2周随访1次，诱导治疗中后期每1～2个月随访1次。

（2）维持治疗期间每3个月随访1次，患者病情稳定后可逐渐延长至每3～6个月随访1次。

5. 随访/回访记录的整理与保存：在门诊档案/患者卡片中增加随访记录，随访内容可包括随访时间、疗效评估和不良事件等。

四、患者教育

建议患者教育团队包含主诊医师及护理团队成员。患教团队应基于最新的医学研究与指南，确保教育材料准确无误且通俗易懂。注意提高患者及家属的参与度、互动性，并提供活动后反馈，跟进支持途径，以增加患者及家属对疾病知识、用药管理等方面的了解。
